# In Situ Electrodeposition of Pb and Ag Applied on Fluorine Doped Tin Oxide Substrates: Comparative Experimental and Theoretical Study

**DOI:** 10.3390/ma15248865

**Published:** 2022-12-12

**Authors:** Ahmed Rebey, Ridha Hamdi, Imen Massoudi, Bechir Hammami

**Affiliations:** 1 Department of Physics, College of Science, Qassim University, Buraidah 51452, Saudi Arabia; 2 Department of Physics, College of Science, Imam Abdulrahman Bin Faisal University, P.O. Box 1982, Dammam 31441, Saudi Arabia; 3 Basic and Applied Scientific Research Center, Imam Abdulrahman Bin Faisal University, P.O. Box 1982, Dammam 31441, Saudi Arabia; 4 Department of Chemistry, College of Science, Qassim University, Buraidah 51452, Saudi Arabia

**Keywords:** lead, silver, in situ electrodeposition, impedance spectroscopy, X-ray diffraction FTO

## Abstract

A comparison between lead and silver electrodeposition onto fluorine-doped tin oxide (FTO) electrodes from nitrate solution was investigated in this work. Chronoamperometry has been used as an in situ technique to track the dynamics of the electrodeposition during advanced nucleation phases. The experimental results are correlated with a theoretical evaluation. It has shown that they have a strong correlation with each other. After that, the obtained deposits are characterized and compared as well by X-ray diffraction (XRD), energy dispersive X-ray analysis (EDX), scanning electron microscopy (SEM), and impedance spectroscopy. The data reflects the effect of the material under investigation on current density, deposition density, and dielectric properties. Additionally, the electrodeposition approach (a two-in-one technique) can be followed in order to make well-controlled thin films that can be used for various purposes in addition to recovering heavy metals from wastewater.

## 1. Introduction

Water is considered the main pathway that leads heavy metals to enter biological systems, and this helps bone and soft tissue cling to it. As a result, harmful damage is affecting not only ecosystems but also humans, animals, and plants. These changes include as well physiologic, genetic, and biochemical changes. Additionally, the different industrial companies all over the world are causing the most environmental harm. Therefore, the primary task which is facing researchers nowadays is to create new methods in order to purify the wastewater produced by various sectors of industry. In the literature, several techniques have been developed. Among them is the activated carbon technique [1], which is associated with several drawbacks as an absorbent. In addition to its high cost, it is necessary to note the fact that it is hard to remove it from water after the process of purification. Moreover, elevated pH leads metals to precipitate [2]. On the other hand, several biological, biochemical, and other technologies are becoming popular in this discipline, referring to examples [3,4,5]. Furthermore, the electrodeposition method represents a compelling technology that has proved its efficiency in the process of extraction of heavy metals [6]. In addition, it is considered a straightforward method with a low-cost and easily scalable method compared to the industrial scale, and it can be used in a variety of settings to control the dimensions and shapes of the deposits [6,7]. Additionally, the deposits extracted from wastewater can be used for other purposes such as sensors, photovoltaic cells, data storage, and different other aims. In comparison to other heavy metal deposits, lead and silver possess distinctive chemical and physical properties [7,8] as they have a long lifespan and they represent resistance to corrosion factors. Moreover, these characteristics open up a lot of possibilities for using lead and silver deposits for various purposes in microelectronics applications, as an example [9,10]. More specifically, when isolated Pb or Ag particles grow from the nanometer to the micrometer scale, their electronic properties change dramatically. Due to their chemical and physical qualities, Pb or Ag are commonly used materials for display devices and for sensor preparation, respectively. Subsequently, industrial waste can be used in interesting technologies.

In this study, we are comparing the electrodeposition of lead and silver from an aqueous solution that produces a thin layer on an FTO substrate. An in situ chronoamperometry and a cyclic voltammetry measurement were used and followed by a theoretical evaluation to support the experimental results. The impedance spectroscopy was used to better understand the propagation of the cation. Furthermore, scanning electron microscopy, energy dispersive X-ray analysis, and X-ray diffraction techniques were used to characterize and compare the deposits. The lead deposit could be an interesting candidate for electrochromic displays, while the silver deposits could be used as a sensor.

## 2. Materials and Methods

**Figure 1:**
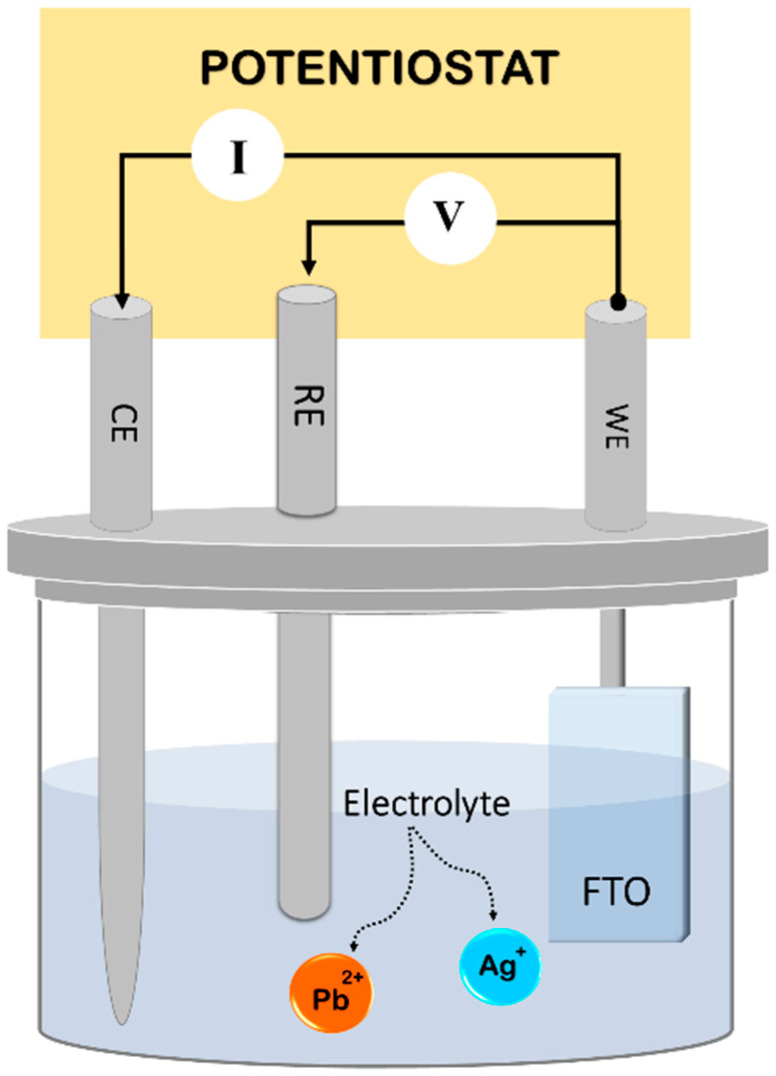
Electrodeposition scheme of the electrodeposition workstation: WE; working electrode, RE; reference electrode, CE; counter electrode.

In order to imitate polluted water, 0.4 M of sodium nitrate (${\mathrm{NaNO}}_{3}$) and 0.5 M of silver nitrate (${\mathrm{AgNO}}_{3}$) or lead nitrate ($\mathrm{Pb}{\left({\mathrm{NO}}_{3}\right)}_{2}$) were employed as electrolytes and nutrients, respectively. They were dissolved in distilled water at 60 °C. After that, ${\mathrm{AgNO}}_{3}$ or $\mathrm{Pb}{\left({\mathrm{NO}}_{3}\right)}_{2}$ was mixed with ${\mathrm{NaNO}}_{3}$ in a molar ratio of 1:3. The FTO substrate used (from Ossila) measures 20 mm by 15 mm. The electrodeposition area was fixed at 10 mm by 15 mm. FTO conducting glass substrates with a resistance of 8 Ω/square were used as electrodeposition substrates. To stir the solution for diffusion, magneton was added during the electrodeposition process. For all measurements made throughout our study, the spacing had been set at 10 mm. The pH of the solution also had a significant impact on the current density and all other physicochemical parameters. Therefore, it was adjusted to be 5.4 as well for all measurements. Several cleaning methods were used before the deposition of FTO substrates, including detergent, distilled water, acetone, and isopropyl alcohol. The electrodeposition experiments were conducted in an open cell with three electrodes (Figure 1). FTO substrates were fixed in the working electrode (WE), and platinum wires were used as counter electrodes (CE). The reference electrode (RE) consists of an Ag/AgCl electrode (Thermo ScientificTM OrionTM, Selangor, Malaysia) in contact with the solution through a Luggin capillary. PalmSens4 (from Palmsen, Houten, The Netherlands) was used for voltammetry, amperometry, and impedance measurements.

## 3. Results and Discussion

### 3.1. Cyclic Voltammetry

**Figure 2:**
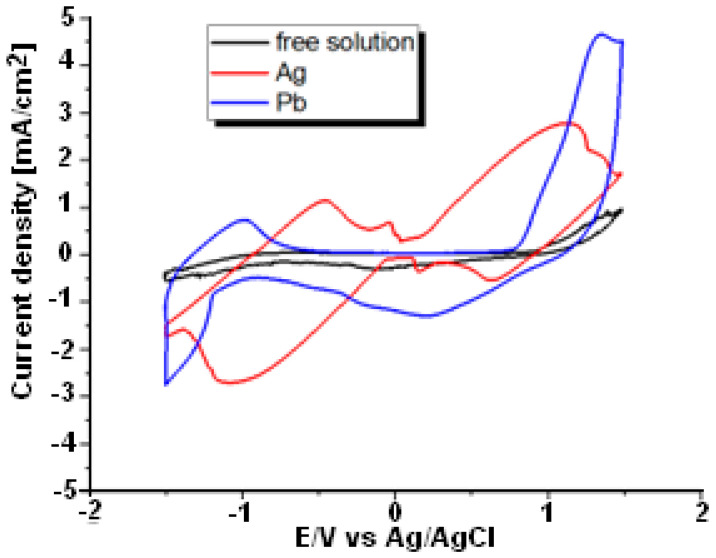
Cyclic voltammogram: black color free solution, red and blue colors presence of Ag and Pb in an aqueous solution, respectively. Scan speed is 60 mV/s.

The deposition potential of Ag or Pb is estimated using cyclic voltammetry (I-V), a static diagram of current I versus voltage V. An I-V curve recorded between −1.5 V and +1.5 V at a change rate of 60 mV/s is shown in Figure 2. Oxidation reactions produce shoulders in the anode leg of both metals as the potential moves in the direction of more positive values. When the potential reaches a predetermined switching potential, a reduction reaction takes place, which causes the direction of the scan to change. This reveals the cathode branch’s shoulder. The (I-V) cycle is used to describe the potential ranges connected to various electrochemical processes. The voltammograms have been thoroughly examined and published elsewhere [11,12]. The outcomes of the experiments are compared. The reference I-V cyclic voltammogram for Ag^+^ and Pb^2+^ free solutions is established (black curve) and shown in Figure 2. The current density on this cyclic is nearly zero (j = 0 mA/cm^2^), and there is no peak in current density. Nevertheless, aqueous solutions of Ag^+^ and Pb^2+^ seemed to affect the current density measured through the voltage window and were presented by the red and blue curves (Figure 2). This can be explained based on Equation (1):

(1) $$M^{n+}\left(aq\right)+\;\;n\;e^{-}\;\;\;\;\to \;\;\;M\;\left(s\right)$$

An external current source (Palmsen4) provides the charge. It appears that the peak position of the current for both metal Ag (red curve) and Pb (blue curve) is generally close to −1 V. However, the peak intensity varies as heavy metal is varied. It is important to note that current stability during repeated potential cycling illustrates the reproducibility of metal deposition processes.

### 3.2. Chronoamperograms Analysis

**Figure 3:**
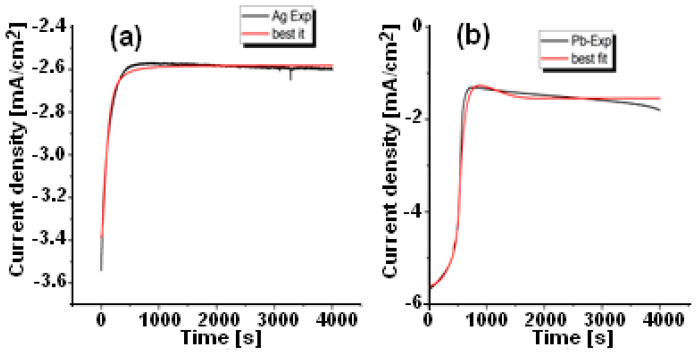
(**a**) Ag and (**b**) Pb electrodeposition current density versus time and their best fit according to Equation (2).

The determination of the microstructure of deposited films and their physicochemical properties can be established through the current density [6,7]. To understand this mechanism properly, it is necessary to understand, compute, and discuss these experimental results. Indeed, as reported by González et al. [13], electrochemical deposition is a very complex and highly condition-dependent process. Accordingly, to simplify this mechanism, one can show two distinct parts that describe the entire process of the chronoamperograms curves in Figure 3, which depict the in situ evolution of the current density. In the first section, the material under test (Pb and Ag) has a significant impact on its duration. It is characterized by a high slope where the current density starts to rise significantly. Whether existing nuclei grow or new nuclei form at the surface of FTO, this section discusses the nucleation process. The Section 2 is where the current density is essentially constant; one could say that both Pb and Ag deposits across the FTO surface reach their equilibrium. It indicates that Pb and Ag have diffused onto the FTO surface. Popov et al. [14] proposed a theory in which they explained that any deposition on any surface could be classified into two scenarios: instantaneous process and progressive process. Hence, the experimental results shown in Figure 3 are fitted with a discussed equation to comprehend the deposition mechanism of Pb as well as Ag on the FTO substrate.

The nucleation process is typically classified into three modes [15], and the growth processes of the current density are modeled by the combination of several equations. The Volmer--Weber model describes it as progressive nucleation and 3D growth. Equation (2) is the next step in the modeling process.

(2) $$J\left(t\right)=i_{0}exp\left(-P_{1}t^{3}\right)+P_{2}\left[1-exp\left(-P_{1}t^{3}\right)\right]$$

where:

$$i_{0}=zFk_{0}$$

$$P_{2}=zFk^{\prime }$$

$$P_{1}=\frac{\pi M^{2}k^{2}A_{3D}}{3\rho^{2}}$$

where J(t) is the current density and t is the time. The atomic weight of the deposit (g mol^−1^) and the density (g cm^−3^) are given by *M* and ρ. They are 107.87 and 10.49 for silver, respectively, and 115.87 and 11.39 for lead, respectively. A3D is the nucleation rate, and *k* and *k*′ are the growth rate constants. The constant *k*_0_ represents the growth rate in the base plane. Assuming the growth rates in both directions are equal, we obtained *k*_0_, *k*′, and A3D by fitting equations. The simulation results are summarized in Table 1. The obtained results are in agreement with what is in the literature [7,16].

### 3.3. Impedance Spectroscopy

**Figure 4:**
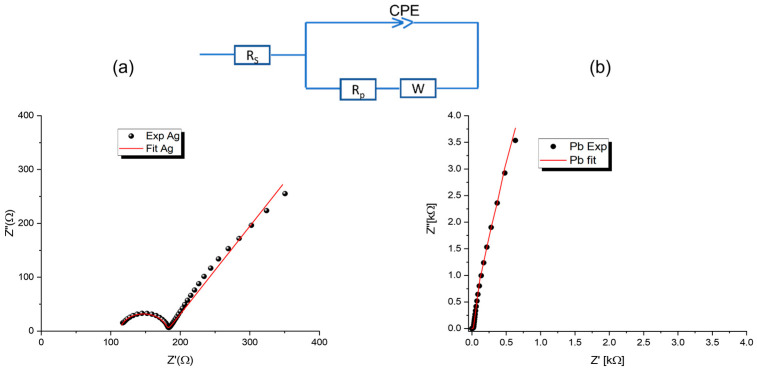
The electrical equivalent circuit for the sample being tested is shown in the inset, along with the associated Nyquist plots. The red line represents the best fit based on this circuit, while the scatter points represent the experimental results (**a**) Silver and (**b**) Lead.

**Figure 5:**
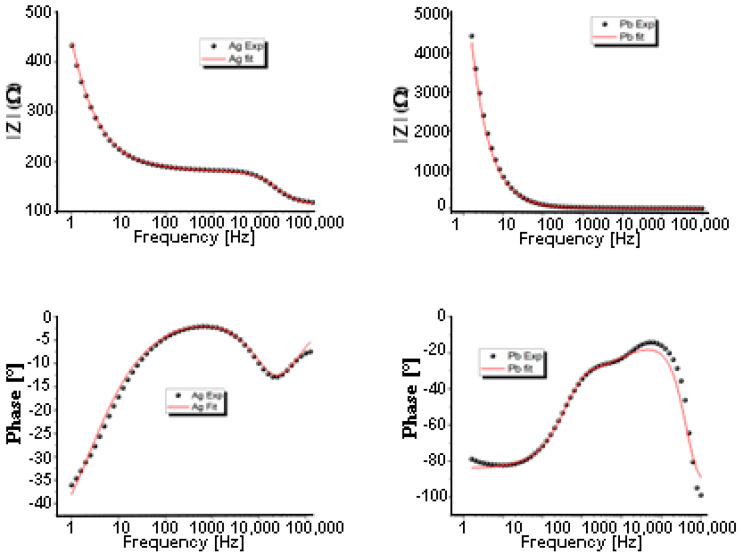
Bode plots: scatter points present the experimental magnitude Bode plot and phase angle Bode plot for lead and silver. Red line represents their best fit based on the equivalent circuit shown in Figure 4.

The complex impedance spectroscopic (CIS) approach is used to compare the electrical response of Ag and Pb in a broad frequency range [1 Hz, 1 MHz]. Figure 4 and Figure 5 show their Nyquist and bode plots, respectively. For Pb, in the 1.0–10 Hz interval, no characteristic frequency can be identified from the Bode plots (Figure 5). Ag, on the other hand, shows significant changes in phase angles. These constant phase elements (CPEs) can be attributed to irregular lead electrodeposition. Furthermore, Nyquist plots (Figure 4) show a slight distortion of the semicircle at high frequencies, followed by a second contribution at intermediate frequencies. Then, at lower frequencies, the Warburg-type straight line is seen. It represents the diffusion of ions into the electrode related to Warburg impedance. Changing from Pb to Ag resulted in more values that are negative and enlarged diffusional impedances. In the CIS spectrum, it is worth noting that in the dynamic response, both the real and imaginary parts of the impedance of Pb were seen to be about 10 times higher than of Ag.

The corresponding electrochemical parameters are determined by matching the features of CIS spectra with an equivalent electrical circuit (inset of Figure 4) containing appropriate elements using ZVIEW software 2.0. The Nyquist shows a Warburg-type straight line in the low-frequency region and a semicircle in the high and medium frequencies region and medium-frequency region. Responses to this experiment can be attributed to the formation of an electric dipole resulting from a series connection of solution resistances (Rs) and capacitors parallel to polarization resistances (Rp). Due to the irregular shape of the scattered points, combining only resistance and a capacitor element is not sufficient to explain CIS results with accuracy. Constant Phase Elements (CPEs) in parallel with Warburg impedance (W) are usually regarded as better representations of the circuit-fitting parameters than constant phase elements in series [17,18,19]. The scattered points in Figure 5 represent experimental results, while the red curve represents their best fit. According to Figure 4 and Table 2, the Rp for silver (68 Ω) is higher than the Rp for lead (~6 Ω). This variation can be explained easily by monitoring the current density in the chronoamperograms analysis shown in Figure 3, where the current density of silver is nearly constant at 2.6 mA/cm^2^ while the current density of lead is about 1.6 mA/cm^2^. The n values for both metals are close to the unit (n = 0.96 for Pb and 0.95 for Ag). The capacity of lead, 6.09 × 10^−10^ F, is three orders less than the capacity of silver, 1.90 × 10^−7^ F.

### 3.4. SEM Analysis

**Figure 6:**
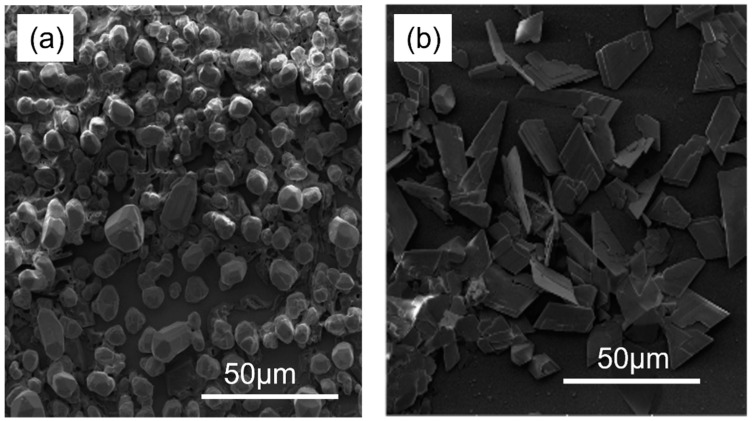
SEM images of (**a**) Ag and (**b**) Pb deposed on FTO substrate.

Electrodeposited Ag (Figure 6a) and Pb (Figure 6b) onto FTO were imaged using SEM to support theoretical results and to analyze surface morphology. Images were analyzed using a JSM-JEOL 6390 Scanning Electron Microscope. The particle size distributions were made with image analyzer software (ImageJ, WRNIH, USA). The particles size distributions were obtained from dark field SEM images by measuring the mean size of more 50 diffracting particles. Initially, the deposit may be significantly larger than subsequent deposits because nucleation does not occur instantly over the entire surface, as previously shown. As a comparison, deposits are more likely to form on surfaces with atomically flat atoms than on those with higher coordination numbers. Nucleation of Ag or Pb on flat surfaces takes longer, which is consistent with the fact that they take longer to form. Approximately the same size rocks are found in the Ag deposits collected. Ag electrodeposits, such as granules, can develop during Ag deposition.

**Figure 7:**
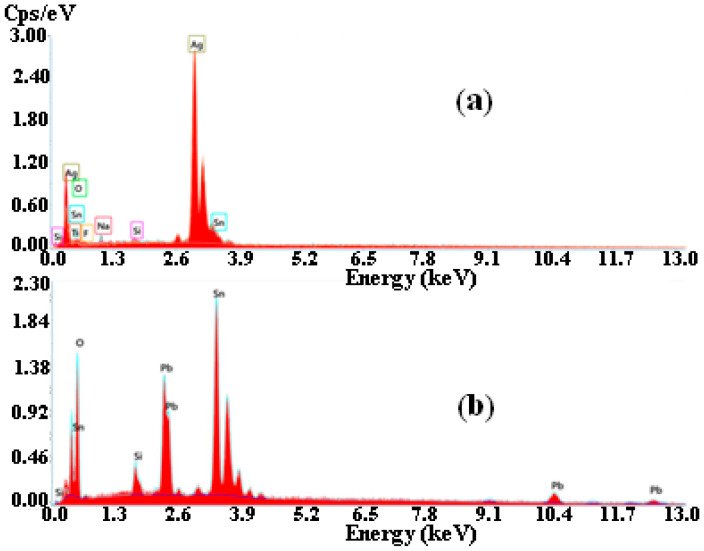
Energy dispersive X-ray analysis spectrum of (**a**) silver and (**b**) lead deposit on FTO substrate.

The silver particles exhibit approximately an identical shape, indicating that silver ions from the surrounding solution are reduced on their surfaces. Tsui et al. [20] and Popov et al. [14] also reported similar findings. In contrast, the morphologies and densities of Pb deposits differ significantly from those of Ag deposits. A sand-rose shape characterizes their morphology, with flat faces and sharp corners. Analyzing chronoamperometry data confirmed the morphologies determined by surface morphology. A number of reports in the literature describe similar morphologies [21,22]. Quantitative analysis of the obtained film is carried out using energy dispersive X-ray analysis. Results presented in Figure 7 show the presence of Pb, Ag, O, Si, Na, and Sn. The presence of Ag (Figure 7a) and Pb (Figure 7b) are the results of the electrodeposition process.

**Figure 8:**
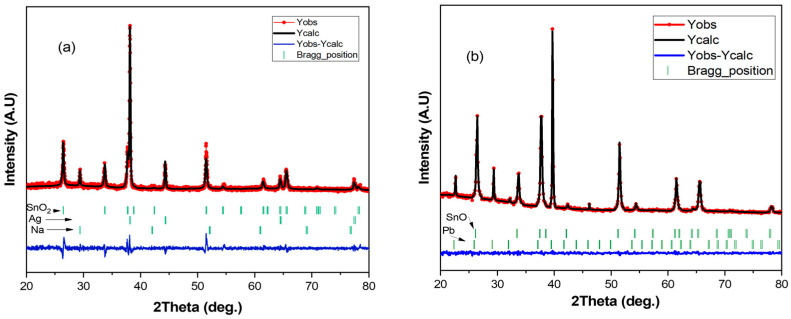
Plots of the Rietveld refinement given the comparison of the experiment with the calculated XRD patterns of (**a**) Ag and (**b**) Pb.

Figure 8 shows the X-ray diffraction patterns of experimental Pb and Ag and their Rietveld refinement results. The red point line is the observed intensities, and the black line is the Rietveld fit. The small vertical green bars display Bragg positions of HKL reflections. The difference between the observed and the calculated XRD patterns is plotted below the diffraction pattern in blue color. The results of the Rietveld refinements show that both silver and lead have a cubic system in Bravais lattices with a = 4.0862 nm and a = 6.8057 nm, respectively. For the deposit of thin film silver, there are three phases: Ag, SnO_2_, and Na. Ag peaks were seen at 38.11, 44.43, 64.40, and 77.41 with corresponding hlk (111), (200), (220), and (311), respectively, with increasing 2θ angle from 20° to 80°. For the deposit of thin film lead, there are two phases: Pb and SnO. At the same interval of angle 2θ, Pb peaks were observed at 26.41, 33.69, 37.70, 42.45, 51.44, 54.37, 61.44, 65.5, and 78.12. The corresponding hlk were (110), (101), (200), (210), (211), (220), (310), (301), and (321), respectively.

## 4. Conclusions

To deposit heavy metals like lead and silver, an FTO substrate was chosen, thereby producing a thin coating that is not uniform and has a variable Pb and Ag density. Using an electrochemical technique, the deposition was created by reducing Pb^2+^ and Ag^+^ cations at a potential of −0.8 V. It was shown how the in situ evolution of the current density changed throughout the deposition processes. To comprehend if new nuclei are formed at the FTO surface or if existing nuclei expand, a mathematical fitting is performed after the findings of the experimental measurement. An SEM was used to examine the surface morphology of the deposits. Ag deposits are demonstrated to have boulder-like shapes roughly the same size, but Pb deposits have sand-rose structures with flat faces and sharp corners. With the help of an EDX in conjunction with SEM, a quantitative analysis of the deposits was performed. Both silver and lead exhibit cubic systems in Bravais lattices, according to the X-ray diffraction patterns of the deposits and their Rietveld refinement results. Additionally, the formation of thin film silver exhibits three phases. However, there are just two phases for the thin film lead deposit.

## Figures and Tables

**Table 1:** Lateral and vertical growth rates and nucleation rates constant values.

Parameters	Pb	Ag
log *k*_0_, mol cm^−2^ s^−1^	−3.537	−3.801
log *k*′, mol cm^−2^ s^−1^	−4.331	−4.887
log *A*_3*D*_, nuclei cm^−2^ s^−1^	10.600	11.126

**Table 2:** Fitting parameters of the equivalent circuit.

	Pb	Ag
RS (Ω)	0.393	112.943
Rp (Ω)	6.1334	67.992
CPE-T (F)	6.094 × 10^−10^	1.9075 × 10^−7^
CPE-P	0.967	0.959
W-R (Ω)	40.68	0.1051
W-T (F)	69.089 × 10^−5^	7.078 × 10^−7^
W-P	0.467	0.325
